# JuNkIE-CLImax: exploring multidimensional images in notebooks and the terminal

**DOI:** 10.17912/micropub.biology.002120

**Published:** 2026-04-02

**Authors:** Rodrigo Fernandez-Gonzalez, Raymond Hawkins

**Affiliations:** 1 Institute of Biomedical Engineering, University of Toronto, Toronto, ON, CA; 2 Ted Rogers Centre for Heart Research, Translational Biology and Engineering Program, University of Toronto, Toronto, ON, CA; 3 Department of Cell and Systems Biology, University of Toronto, Toronto, ON, CA; 4 Developmental and Stem Cell Biology Program, Hospital for Sick Children, Toronto, ON, CA

## Abstract

Modern microscopy generates multidimensional images. Graphical user interfaces allow visualization of multidimensional images, but image exploration is significantly harder in text-based environments. These include Jupyter notebooks, popular for the distribution of data analysis pipelines; and the terminal, often the only user interface available when accessing images in remote servers. We developed JuNkIE-CLImax, a pair of multidimensional image explorers designed to work in Jupyter notebooks and the terminal, respectively. JuNkIE-CLImax are written in Python, using Rust extensions to optimize performance; they are platform agnostic; and they provide application programming interfaces that enable integration into complex image visualization and analysis pipelines.

**Figure 1. JuNkIE-CLImax: multidimensional image explorers for notebooks and the terminal:** **(A)** JuNkIE (top) and CLImax (bottom) user interfaces displaying multidimensional images in a notebook and the terminal, respectively. JuNkIE-CLImax provide widgets to browse different image dimensions; expose multiple colormaps, including scientific colormaps; and conduct simple image operations, including rotation, flipping, zooming, reslicing and contrast adjustment. **(B)** Character-based image rendering. Sample image (left) and corresponding *rich* Style strings to render the image in the terminal (right), using a half-block character to display each pair of vertically-adjacent pixels. For instance, the Style parsed from " *rgb(255,0,0) on rgb(0,255,255)* ", when associated with the half-block character, renders a red half-block on a cyan background, representing a cyan pixel in the top row and a red pixel in the bottom row, respectively *.* End-of-line characters ("\n") indicate the ends of pairs of rows.

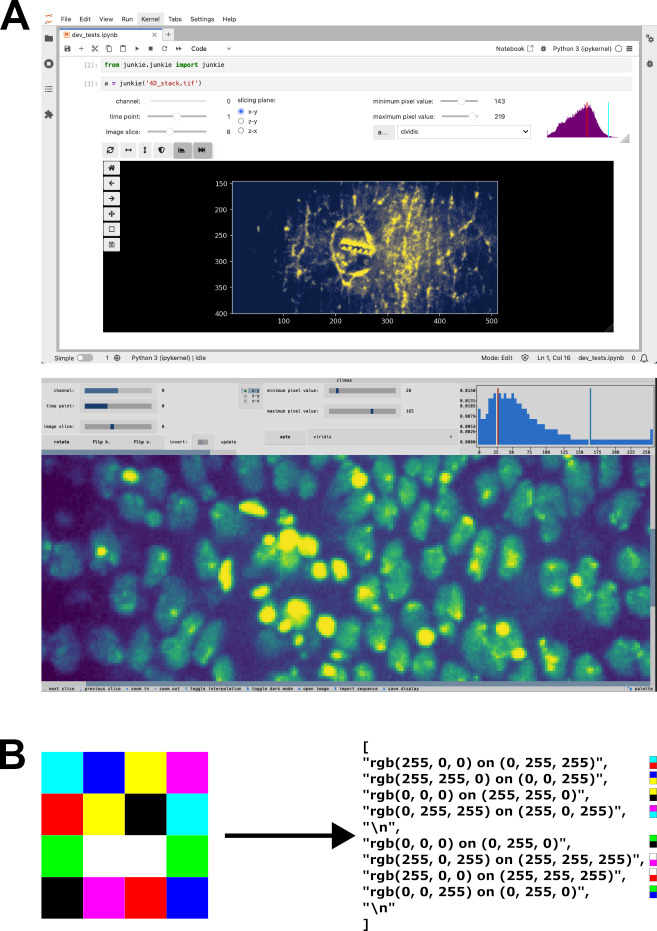



## Description

Quantitative image analysis of microscopy datasets is a central tool in modern cell and developmental biology (Meijering and Cappellen, 2007; Senft et al., 2023). The advent of machine learning methods has established Python as the *de facto* standard for the analysis of biomedical images (Jacquemet, 2021; Morgado et al., 2024). Jupyter notebooks (Granger and Pérez, 2021) provide a beginner-friendly environment that enables prototyping and distribution&nbsp;of complex image processing pipelines (von Chamier et al., 2021). Platforms such as Google Colab or DigitalOcean provide free access to high-end computing capacity to execute notebooks, thus "democratizing" the use of resources that would otherwise be too costly. Notebooks facilitate data exploration and increase the reproducibility of data processing pipelines (Kluyver et al., 2016; Pimentel et al., 2019; Samuel and Mietchen, 2024). The leading position of notebooks for data analysis is cemented by the recent availability of large language models within notebooks (Qiu, 2023), which further facilitates development, debugging and execution of image processing and quantification routines. Thus, notebooks are a central element in the analysis of biomedical image data.

Both biological and medical imaging generate multidimensional data. Confocal and light sheet microscopies, for instance, allow collection of three-dimensional stacks (X, Y, Z) of different fluorescent reporters (wavelength), over the duration of a biological process (time). Multimodality imaging, such as correlative confocal and electron microscopies (Casares-Arias et al., 2021), confocal and super-resolution (Xiang et al., 2018), or the combination of anatomical and functional data in medical imaging (Pichler et al., 2008), introduces a sixth imaging dimension. Processing and analysis of multidimensional image datasets often requires image exploration at many stages in which rapid visualization and browsing are key steps. Two-dimensional images can be easily displayed in notebooks, *e.g.* with *matplotlib* (Hunter, 2007). However, few tools exist to display, browse and interact with multidimensional images in a notebook (Haase, 2021), and some of the available options provide complex user interfaces and functionality that prevent light-weight, inline multidimensional image browsing (Sofroniew et al., 2025).

Microscopy images are generally archived in remote backup servers. The advent of cloud-based storage services has only increased this trend.&nbsp;Remote and cloud-based servers often do not provide a graphical user interface, but simply a terminal-based, command line interface. Terminals can display text-based information about the images (file name, size, date, etc.), but do not allow image visualization. The lack of tools for image display on the terminal makes it hard to select images either for further processing or to generate figures for publication.

Here we introduce JuNkIE-CLImax, a pair of open source tools (https://bitbucket.org/rfg_lab/junkie and https://bitbucket.org/rfg_lab/climax) for the display and interactive visualization of multidimensional microscopy images in environments traditionally considered to be document or text-based. JuNkIE is a JUpyter NotebooK Image Explorer that enables the opening, display and simple manipulation of multidimensional images in Jupyter notebooks. CLImax, a Command Line IMAge eXplorer, allows users to open, visualize and browse multidimensional images on the terminal, using character-based strategies for image rendering.

JuNkIE-CLImax: multidimensional image explorers

JuNkIE-CLImax can load multidimensional images in the multi-page TIFF format often found in microscopy (Besson et al., 2019), as well as images stored in the increasingly popular *zarr* array format (Miles, 2015). JuNkIE-CLImax can also import file sequences that represent different dimensions ( *e.g.* time or wavelength) of a multidimensional image. Internally, images are stored using *numpy* , a package for numerical computation, and its *ndarray* data structure to represent multidimensional arrays (Harris et al., 2020). JuNkIE-CLImax display a two-dimensional view of a multidimensional image ( Fig. 1A ). Both packages provide sliders to select a specific Z-plane, time point and channel from the multidimensional image stored in memory, using both mouse and keyboard-based interactions. JuNkIE-CLImax continuously update the displayed image in real time in response to user input. To enable use in settings with reduced compute power ( *e.g.* remote storage servers), it is possible to disable the continuous update of the display, such that updates only occur when the user interaction is completed ( *e.g.* when the mouse button is released, but not while the mouse is dragged). XY, XZ or YZ sectioning planes can be selected. Additionally, JuNkIE-CLImax provide functionality for simple image manipulation. This includes rotation and flipping (both horizontal and vertical), colormap selection and inversion (including all the colormaps available in *matplotlib* (Crameri et al., 2020; Crameri, 2023)), and both manual and automated contrast adjustment.

***JuNkIE***

JuNkIE displays images using the *Figure* class in *matplotlib* . A *matplotlib* *Figure* can hold different plot elements, including images. The *matplotlib* *Figure* provides a toolbar with options to pan, zoom in and out, reset the view, or save a snapshot ( Fig. 1A, top).

JuNkIE provides a simple application programming interface (API). Using the JuNkIE API it is possible to open images from disk, by providing a file or folder path, or to view volumetric information already loaded as an *ndarray* . Substrings can be used to distinguish which files in an image sequence store specific dimensions ( *e.g.* different image channels). Lastly, the colormap used to initially display an image, as well as the size of the figure can be determined with input parameters when invoking JuNkIE.&nbsp;&nbsp;&nbsp;&nbsp;

JuNkIE is distributed with a test suite using *pytest* (Krekel et al., 2004) and the *nbval* plugin for notebook-based testing (Cortes-Ortuno et al., 2016), with code coverage over 90%.

***CLImax***

To display images on the terminal, we created a character-based image renderer. The image renderer uses *rich* , a Python package for text formatting that allows the use of coloured characters in the terminal (McGugan, 2019; Burns, 2022). Our image renderer takes a two-dimensional *ndarray* as its input, and produces a sequence of *rich* Segments, each consisting of a character and some formatting (a *Style* ), punctuated by end-of-line characters at the end of each image row ( Fig. 1B ). *rich Styles* determine the colours used to display a character and its background. Colours are defined by the corresponding red, green and blue (rgb) components, specified as a string. Initially, we rendered each pixel using a space character (' '), assigning the pixel value as the background colour and no foreground colour. Because characters on the terminal are rectangular (elongated along the vertical axis), we rendered each image column twice to obtain square pixels. As a consequence, image rendering was relatively slow, requiring 380±23 ms (mean±standard deviation) to render a 512x672 pixel image on an Apple M3 Max equipped with 48 GB of RAM.

To optimize image rendering, we first reduced the number of terminal characters necessary to display a pixel. We took advantage of the half-block character ('▄') to visualize pairs of vertically-adjacent pixels (same column, consecutive rows) with a single terminal character ( Fig. 1B ). For each pair of pixels, we rendered the bottom pixel by assigning its value as the foreground colour of a half-block character, and the top pixel by assigning its value as the background colour of the same half-block character ( Fig. 1B ). This approach allowed us to display each pixel with half a character, in contrast to the previous method, which required two characters per pixel. We further accelerated rendering by creating a *Style* cache that used a Python dictionary to store the *rich* *Styles* corresponding to parsed colour-strings. After these optimizations, rendering speed increased by 57% (163±13 ms/image) and memory consumption decreased by a factor of four.

To further speed up image display, we implemented the hot-loop of the renderer in Rust (Matsakis and Klock II, 2014). The Rust code implements a helper function with two inputs, a two-dimensional image, and a colour lookup table that contains the string representation of each of the colours available in the current colormap. The helper function then scans the image columns, two rows at a time, generating the *Style* string for the character that will display a pair of vertically-adjacent pixels. We parallelized the scan over the image columns using the Rust crate *rayon* (Matsakis and Stone, 2014) to create a parallel iterator. Overall, Rust-based image rendering was 11% faster (146±13 ms/image) than our fastest Python implementation.

We designed the CLImax user interface with *textual* (McGugan, 2021) ( Fig. 1A, bottom). *textual* is a Python package for the development of user interfaces and applications for the terminal. *textual* provides different widgets ( *e.g.* buttons, dropdown boxes, text labels, etc.). We created a new *ImagePanel* widget that displays an image using the character-based renderer described above. *textual* apps provide a command palette that includes options to change the theme used to display the application, save a screenshot, or show a help panel. In addition, CLImax provides options to open a new image, zoom in or out, display the image with bilinear interpolation (particularly useful when zooming in/out), or save the current display.

CLImax can be invoked from the command line with different options. Similar to the JuNkIE API, the CLImax command line parameters enable image selection and substring and colour map specification, as well as initial zoom level, particularly important given that image rendering in the terminal is computationally intensive, with a cost that increases with the number of pixels to be rendered.

CLImax is also tested with *pytest* , using the *asyncio* plugin (Seifert, 2015) to test asynchronous user interaction. CLImax tests provide code coverage greater than 90%.

## References

[R1] Besson Sébastien, Leigh Roger, Linkert Melissa, Allan Chris, Burel Jean-Marie, Carroll Mark, Gault David, Gozim Riad, Li Simon, Lindner Dominik, Moore Josh, Moore Will, Walczysko Petr, Wong Frances, Swedlow Jason R. (2019). Bringing Open Data to Whole Slide Imaging. Lecture Notes in Computer Science.

[R2] Burns,D. (2022) rich-pixels, https://github.com/darrenburns/rich-pixels.

[R3] Casares-Arias Javier, Alonso Miguel A., San Paulo Álvaro, González María Ujué (2021). Correlative confocal and scanning electron microscopy of cultured cells without using dedicated equipment. STAR Protocols.

[R4] von Chamier Lucas, Laine Romain F., Jukkala Johanna, Spahn Christoph, Krentzel Daniel, Nehme Elias, Lerche Martina, Hernández-Pérez Sara, Mattila Pieta K., Karinou Eleni, Holden Séamus, Solak Ahmet Can, Krull Alexander, Buchholz Tim-Oliver, Jones Martin L., Royer Loïc A., Leterrier Christophe, Shechtman Yoav, Jug Florian, Heilemann Mike, Jacquemet Guillaume, Henriques Ricardo (2021). Democratising deep learning for microscopy with ZeroCostDL4Mic. Nature Communications.

[R5] Cortes-Ortuno,D. *et al.* (2016) nbval, https://github.com/computationalmodelling/nbval.

[R6] Crameri Fabio (2023). Scientific colour maps.

[R7] Crameri Fabio, Shephard Grace E., Heron Philip J. (2020). The misuse of colour in science communication. Nature Communications.

[R8] Granger Brian E., Perez Fernando (2021). Jupyter: Thinking and Storytelling With Code and Data. Computing in Science & Engineering.

[R9] Haase,R. (2021) stackview, https://github.com/haesleinhuepf/stackview.

[R10] Harris Charles R., Millman K. Jarrod, van der Walt Stéfan J., Gommers Ralf, Virtanen Pauli, Cournapeau David, Wieser Eric, Taylor Julian, Berg Sebastian, Smith Nathaniel J., Kern Robert, Picus Matti, Hoyer Stephan, van Kerkwijk Marten H., Brett Matthew, Haldane Allan, del Río Jaime Fernández, Wiebe Mark, Peterson Pearu, Gérard-Marchant Pierre, Sheppard Kevin, Reddy Tyler, Weckesser Warren, Abbasi Hameer, Gohlke Christoph, Oliphant Travis E. (2020). Array programming with NumPy. Nature.

[R11] Hunter John D. (2007). Matplotlib: A 2D Graphics Environment. Computing in Science & Engineering.

[R12] Jacquemet Guillaume (2021). Deep learning to analyse microscopy images. The Biochemist.

[R13] Kluyver Thomas, Ragan-Kelley Benjamin, P&eacute;rez Fernando, Granger Brian, Bussonnier Matthias, Frederic Jonathan, Kelley Kyle, Hamrick Jessica, Grout Jason, Corlay Sylvain, Ivanov Paul, Avila Dami&aacute;n, Abdalla Safia, Willing Carol, Jupyter Development Team (2016). Jupyter Notebooks &ndash; a publishing format for reproducible computational workflows. Positioning and Power in Academic Publishing: Players, Agents and Agendas.

[R14] Krekel,H. *et al.* (2004) pytest, https://github.com/pytest-dev/pytest.

[R15] Matsakis,N.D. and Klock II,F.S. (2014) The rust language. In, *ACM SIGAda Ada Letters* . ACM, pp. 103–104.

[R16] Matsakis,N.D. and Stone,J. (2014) Rayon: A data parallelism library for Rust, https://github.com/rayon-rs/rayon/.

[R17] McGugan,W. (2019) rich, https://github.com/Textualize/rich.

[R18] McGugan,W. (2021) Textual, https://textual.textualize.io/.

[R19] Meijering Erik, Cappellen Gert van (2007). Quantitative Biological Image Analysis. Principles and Practice.

[R20] Miles,A. (2015) zarr, https://github.com/zarr-developers/zarr-python.

[R21] Morgado Leonor, Gómez‐de‐Mariscal Estibaliz, Heil Hannah S., Henriques Ricardo (2024). The rise of data‐driven microscopy powered by machine learning. Journal of Microscopy.

[R22] Pichler Bernd J., Judenhofer Martin S., Pfannenberg Christina (2008). Multimodal Imaging Approaches: PET/CT and PET/MRI. Handbook of Experimental Pharmacology.

[R23] Pimentel Joao Felipe, Murta Leonardo, Braganholo Vanessa, Freire Juliana (2019). A Large-Scale Study About Quality and Reproducibility of Jupyter Notebooks. 2019 IEEE/ACM 16th International Conference on Mining Software Repositories (MSR).

[R24] Qiu,D.L. (2023) Jupyter AI, https://github.com/jupyterlab/jupyter-ai.

[R25] Samuel Sheeba, Mietchen Daniel (2024). Computational reproducibility of Jupyter notebooks from biomedical publications. GigaScience.

[R26] Seifert,M. (2015) pytest-asyncio, https://github.com/pytest-dev/pytest-asyncio.

[R27] Senft Rebecca A., Diaz-Rohrer Barbara, Colarusso Pina, Swift Lucy, Jamali Nasim, Jambor Helena, Pengo Thomas, Brideau Craig, Llopis Paula Montero, Uhlmann Virginie, Kirk Jason, Gonzales Kevin Andrew, Bankhead Peter, Evans Edward L., Eliceiri Kevin W., Cimini Beth A. (2023). A biologist’s guide to planning and performing quantitative bioimaging experiments. PLOS Biology.

[R28] Sofroniew Nicholas, Lambert Talley, Bokota Grzegorz, Nunez-Iglesias Juan, Sobolewski Peter, Sweet Andrew, Gaifas Lorenzo, Evans Kira, Burt Alister, Doncila Pop Draga, Yamauchi Kevin, Weber Mendonça Melissa, Rodríguez-Guerra Jaime, Liu Lucy, Buckley Genevieve, Vierdag Wouter-Michiel, Anderson Ashley, Monko Timothy, Willing Carol, Royer Loic, Can Solak Ahmet, Harrington Kyle I. S., Abramo Jacopo, Ahlers Jannis, Ajina Sesan, Althviz Moré Daniel, Amsalem Oren, Andò Edward, Annex Andrew, Aronssohn Constantin, Balzaretti Filippo, Boone Peter, Bestak Kresimir, Bragantini Jordão, Bunten David, Bussonnier Matthias, Caporal Clément, Chazotte Margot, Coccimiglio Ian, Čočková Zuzana, Eglinger Jan, Eisenbarth Andreas, Freeman Jeremy, Fukai T. Yohsuke, Gohlke Christoph, Gunalan Kabilar, Halchenko Yaroslav Olegovich, Har-Gil Hagai, Harfouche Mark, Hilsenstein Volker, Hutchings Katherine, Kawai Hiroki, Kozar Robert, Lauer Jessy, Le Meur-Diebolt Samuel, Lichtner Gregor, Liu Hanjin, Liu Ziyang, Lowe Alan, Marconato Luca, Martin Sean, McGovern Abigail, Migas Lukasz, Miller Nadalyn, Miñano Sofía, Muñoz Hector, Müller Jan-Hendrik, Nauroth-Kreß Christopher, Obenhaus Horst A., Palecek David, Pape Constantin, Perlman Eric, Theart Rensu Petrus, Pevey Kim, Peña-Castellanos Gonzalo, Pierré Andrea, Pinto David, Rodríguez-Reza Carlos M., Ross David, Russell Craig T., Ryan James, Selzer Gabriel, Smith MB, Smith Paul, Sofiiuk Konstantin, Soltwedel Johannes, Stansby David, Vanaret Jules, Wadhwa Pam, Weigert Martin, Windhager Jonas, Winston Philip, Yu Qin, Zhang Liudeng, Zhao Rubin, Witz Guillaume, Leomil Zoccoler Marcelo (2026). napari: a multi-dimensional image viewer for Python.

[R29] Xiang Wanqing, Roberti M. Julia, Hériché Jean-Karim, Huet Sébastien, Alexander Stephanie, Ellenberg Jan (2018). Correlative live and super-resolution imaging reveals the dynamic structure of replication domains. Journal of Cell Biology.

